# A Toolbox for the Determination of Nitroaromatic Explosives in Marine Water, Sediment, and Biota Samples on Femtogram Levels by GC-MS/MS

**DOI:** 10.3390/toxics9030060

**Published:** 2021-03-16

**Authors:** Tobias Hartwig Bünning, Jennifer Susanne Strehse, Ann Christin Hollmann, Tom Bötticher, Edmund Maser

**Affiliations:** Institute of Toxicology and Pharmacology for Natural Scientists, University Medical School Schleswig-Holstein, Brunswiker Straße 10, 24105 Kiel, Germany; buenning@toxi.uni-kiel.de (T.H.B.); strehse@toxi.uni-kiel.de (J.S.S.); hollmann@toxi.uni-kiel.de (A.C.H.); stu207180@mail.uni-kiel.de (T.B.)

**Keywords:** explosives, trinitrotoluene, GC-MS/MS, large volume injection, solid-phase extraction, blue mussel, sediment, dumped munitions, limit of detection, method improvement

## Abstract

To determine the amount of the explosives 1,3-dinitrobenzene, 2,4-dinitrotoluene, 2,4,6-trinitrotoluene, and its metabolites in marine samples, a toolbox of methods was developed to enhance sample preparation and analysis of various types of marine samples, such as water, sediment, and different kinds of biota. To achieve this, established methods were adapted, improved, and combined. As a result, if explosive concentrations in sediment or mussel samples are greater than 10 ng per g, direct extraction allows for time-saving sample preparation; if concentrations are below 10 ng per g, techniques such as freeze-drying, ultrasonic, and solid-phase extraction can help to detect even picogram amounts. Two different GC-MS/MS methods were developed to enable the detection of these explosives in femtogram per microliter. With a splitless injector, limits of detection (LODs) between 77 and 333 fg/µL could be achieved in only 6.25 min. With the 5 µL programmable temperature vaporization—large volume method (PTV-LVI), LODs between 8 and 47 fg/µL could be achieved in less than 7 min. The detection limits achieved by these methods are among the lowest published to date. Their reliability has been tested and confirmed by measuring large and diverse sample sets.

## 1. Introduction

The release of nitro-aromatic explosives from corroding marine-dumped munitions is a worldwide problem with growing environmental concern [1]. In Germany alone, it is assumed that 1.8 million metric tons of conventional munitions were dumped into the North and Baltic Seas during or after the First and Second World Wars, and 1.6 million metric tons of these munitions are still rusting at the seafloor to date [2]. For 2,4,6-trinitrotoluene (TNT), one of the main explosives used in bombs, grenades, mines, and torpedoes, accumulation has been proven in marine organisms, such as the blue mussel *Mytilus edulis* [3,4,5,6] and dab *Limanda limanda* [7,8]. In order to assess contamination of the North Sea and the Baltic Sea by the legacy of war as comprehensively as possible, with regard to present and future ecotoxicological consequences, the examination of large quantities of various abiotic and biotic samples for TNT, its metabolites, and other munitions components such as 1,3-dinitobenzene and 2,4-dinitrotoluene is required.

Gas and liquid chromatography techniques have been routinely used in the detection of nitro-organic explosives for over fifty years [9,10]. Coupled with mass selective detectors, they not only allow quantitative analysis but also qualitative screening for unknown substances or unsuspected compounds in samples. Modern mass spectrometric techniques, such as triple, quadrupole, or Orbitrap mass spectrometry, allow limits of detection, depending on the components, in the range of femto- to atto-gram per injection [11]. Several publications have since been published dealing with the detection of various explosives and their metabolites in quite different matrices, such as the EPA methods 529 (explosives in drinking water by GC/MS) [12], 8330 (water, soil, and sediment by HPLC) [13], and 8095 (explosives by gas chromatography) [14]. Fields addressed in these publications were exposure to legacies of land-based warfare [15], anti-terror [16], or forensic issues such as the detection of minute traces of explosives in evidence such as clothing and containers [17]. However, more than twenty years ago, research groups have investigated the release of explosives into seawater at marine munitions dumping grounds [18]. Regarding the detection of explosives in marine samples, a whole series of articles have been published in recent years, e.g., for water [19], sediment [19,20], blue mussels [3,4,5,6], and dab [7,8].

In abiotic samples, such as water or sediments, larger concentrations of unmetabolized TNT were found, while it is rarely detectable in biota samples, e.g., in the bioindicator mussel *Mytilus* spp. [3]. Here, mainly TNT metabolites such as 4-amino-2,6-dinitrotoluene (4-ADNT), and to a lesser extent, 2-amino-4,6-dinitrotoluene (2-ADNT), were found [3]. Additionally, biota samples contain large quantities of polar organic molecules, such as fatty acids and steroids, the so-called sample matrix, which coelute during the extraction of the explosives and, therefore, interfere with the analytical material. In abiotic samples, the proportion of soluble matrix is usually lower.

In this study, sample preparation methods were developed and optimized for the detection of TNT, its metabolites 2- and 4-ADNT, and the explosive byproducts 1,3-dinitrobenzene (1,3-DNB) and 2,4-dinitrotoluene (2,4-DNT). The goal was to develop a toolbox of methods that enable the detection of these compounds in different abiotic and biotic samples at sub-nanogram levels. Depending on the expected concentrations, many samples can either be processed quickly or purified and concentrated better through additional steps to achieve even lower detection limits.

## 2. Materials and Methods

### 2.1. Materials and Chemicals

Trinitrotoluene (98.9% purity, 1 mg/mL, in acetonitrile (ACN):methanol (MeOH) 50:50), 1,3-dinitrobenzene (97.0% purity, 1 mg/mL, in ACN:MeOH 50:50), 2,4-dinitrotoluene (98.3% purity, 1 mg/mL, in ACN:MeOH 50:50), 4-amino-2,6-dinitrotoluene (98.4% purity, 1 mg/mL, in ACN:MeOH 50:50), and 2-amino-4,6-dinitrotoluene (97.8% purity, 1 mg/mL, in ACN:MeOH 50:50) were purchased from AccuStandard, New Haven, USA. Isotopically labeled TNT (^13^C_7_, 99%; ^15^N_3_, 98%, 1 mg/mL in benzene, wetted with >33% H_2_O) was purchased from Cambridge Isotope Laboratories, Inc., Andover, USA. Acetonitrile (UHPLC-grade, purity ≥ 99.97%) was purchased from Th. Geyer (Renningen, Germany) and used without further purification. CHROMABOND Easy polystyrene-divinylbenzene-copolymer reversed-phase solid-phase extraction columns 80 µm, 3 mL/200 mg (Macherey Nagel, Düren, Germany) were used. Ultrapure water (18.2 MΩ cm) was prepared on-site with a Veolia ELGA Purelab Flex system. Sea water was collected with a 2 L Ruttner water trap (Hydro-Bios, Kiel, Germany) from the Kiel Fjord. Live blue mussels (food grade) were purchased from the Kiel mussel farm and stored at −20 °C before use. Freeze-dried sediments from previous studies, stored at −20 °C, were used.

### 2.2. Water Sample Preparation

Water samples were prepared by a method adapted from Gledhill et al. (2019) [19]. Seawater (1L) was measured into an EVA infusion bag (ICU Medical, Inc, San Clemente, CA, USA) containing 200 µL of a 250 ng/mL ^13^C^15^N-TNT solution as internal standard, and introduced through an unconditioned Chromabond Easy SPE column in the absence of light at 4 °C. The water was then discarded, columns were dried in vacuum (0.5 h) and eluted with 4 mL ACN. The eluate was concentrated to 600 µL using a RVC 2-25 CDplus rotary vacuum concentrator (Martin Christ Gefriertrocknungsanlagen GmbH, Osterode, Germany) and stored in 1.5 mL amber vials at −20 °C.

### 2.3. Sediment Sample Preparation

Twenty grams of wet sediment were weighed into 100 mL screw top beakers and lyophilized in an Alpha 2–4 LSCplus freeze drier (Martin Christ Gefriertrocknungsanlagen GmbH, Osterode, Germany) for 16–18 h. Samples were homogenized by shaking and 2 g of each was weighed into 15 mL polypropylene tubes. ^13^C^15^N-TNT (100 µL of a 250 ng/mL solution) and 4.9 mL ACN were then added, and the samples were shaken on a VF2 vortex mixer (Ika Works Inc., Staufen im Breisgau, Germany) for 60 s, prior to sonication for 15 min (Bandelin Sonorex Super RK 510 H, BANDELIN electronic GmbH & Co. KG, Berlin, Germany). After 10 min of centrifugation (4100 rpm, 4 °C, Heraeus Megafuge 11 R, Thermo Fisher Scientific Inc., Waltham, MA, USA), supernatants were filtered through 0.22 µm PTFE filters, concentrated to 600 µL using a Christ RVC 2-25 CDplus rotary vacuum concentrator and stored in 1.5 mL amber vials at −20 °C.

To achieve lower limits of detection, 100 g wet sediment were mixed with 250 mL bidest water, shaken for 80 min, sonicated for 15 min, centrifuged (4500 rpm at 10 °C for 15 min; J2-HS centrifuge, Beckman Coulter GmbH, Krefeld, Germany), filtered through a 595 1/2 pleated filter and introduced into SPE-columns using mild vacuum. Columns were dried for 30 min i.vac., eluted with 4 mL ACN, concentrated to 600 µL, and stored at −20 °C in 1.5 mL amber vials.

### 2.4. Mussel Sample Preparation

Fresh mussels were prepared according to Strehse et al. 2017 [3]. Mussels were thawed, homogenized using an IKA T25 ULTRA Turrax (IKA Werke GmbH & Co. Kg, Staufen im Breisgrau, Germany) and aliquoted as 1 g portions into 15 mL polypropylene tubes. Five milliliters ACN was added to each sample, and samples were vortexed for 1 min and centrifuged for 10 min (4100 rpm, 4 °C). The supernatants were transferred to 10 mL graded flasks and made up to 10 mL with ACN. One milliliter of each solution was transferred into a 1.5 mL amber vial containing 50 ng ^13^C^15^N-TNT and stored at −80 °C.

For solid-phase extraction, mussels were freeze-dried for 48–72 h. Lyophilized mussels were homogenized using an IKA A11 basic analytical mill (IKA Werke GmbH & Co. Kg, Staufen im Breisgrau, Germany), and aliquots of 150–500 mg were weighed into lightproof 5 mL tubes. An amount of 1.9 mL ACN and 100 µL of a 50 ng/mL ^13^C^15^N-TNT solution were added as internal standard, then samples were vortexed for 60 s, sonicated for 15 min, and centrifuged at 4100 rpm (4 °C) for 15 min. Supernatants were transferred into 25 mL graded flasks, made up with ultrapure water, and introduced onto unconditioned Chromabond Easy SPE-columns using a mild vacuum. Columns were then dried i.v.ac for 30 min, and samples were eluted with 4 mL ACN, concentrated to 600 µL, and stored at −80 °C in 1.5 mL amber vials.

### 2.5. GC-MS/MS Analysis

A Thermo Scientific TRACE 1310 gas chromatograph, equipped with a split/splitless-injector (SSL) and a programmable temperature vaporization (PTV) injector, a TriPlus 100 LS autosampler, and coupled to a TSQ 8000 EVO triple quadrupole mass spectrometer with electron ionization source was used. The GC was equipped with a TraceGold TG-5MS amine 15 m × 0.25 mm × 0.25 µm column (Thermo Fisher Scientific Inc., Waltham, MA, USA) and was connected to the respective injector. Splitless injections on the SSL injector were performed on quartz wool (Thermo Fisher Scientific Inc., Waltham, MA, USA) or CarboFrit (Restek Coorperation, Bellefonte, PA, USA) injection port liner (dimensions: 4 mm × 6.5 mm × 78.5 mm), while large-volume injections were carried out on the PTV-injector with packed quartz wool liners (2 mm × 2.75 mm × 120 mm, Thermo Fisher Scientific Inc., Waltham, MA, USA)). Helium served as a carrier gas for the GC, and Argon as collision gas for the mass spectrometer (both Alphagaz, purity 99.999%). Spectra were recorded and analyzed in TraceFinder 4.1 and Chromeleon 7.2 (Thermo Fisher Scientific Inc., Waltham, MA, USA). GC-MS/MS programs developed for this study are outlined in Table 1.

### 2.6. External and Internal Standards

External standards were prepared by diluting a 100 µg/mL solution of each of the five explosives with ACN to obtain concentrations of 0.1–100 ng/mL. Calibration curves were automatically calculated by the software Chromeleon, based on double injections of a standard row before each batch of samples. During measurements, an external standard in the order of magnitude of the expected concentrations was injected at regular intervals between samples. For TNT, isotopically pure ^13^C^15^N-TNT was used as the internal standard. Solutions of 50, 250, and 500 ng/mL ACN were prepared, and depending on the desired standard concentration, 100 uL of one of the solutions were added to the samples. The signals of both internal and external standards were used to compensate for the change in signal intensity caused by matrix effects over the course of measurements. To check for carry-over effects, a blank solvent was measured before each external standard. Even with concentrations of up to 1000 ng/mL, no carry-over was observed.

### 2.7. Matrix Standards

Matrix-specific limits of detection and quantification were determined with spiked matrix samples. Uncontaminated sediments and mussel samples from previous investigations, as well as water from the Kiel Fjord and 18.2 MΩ cm ultrapure water, were used. From previously freeze-dried sediments, 2 g aliquots were prepared as described above, concentrated to 600 µL, and spiked with all five explosive chemicals at concentrations of 0.1–0.9 ng/mL. Water samples from Kiel Fjord were extracted in the solid phase, concentrated to 600 µL, and spiked in the same manner. Since explosives were found in the Fjord water at concentrations ranging from 0.12–2.2 ng/L, additional ultrapure water was concentrated through Chromabond Easy SPE columns and the eluates were spiked with explosives at concentrations from 0.1 to 0.9 ng/mL of the explosives. Mussels, purchased from the Kiel mussel farm in August 2020, were prepared according to Strehse et al. 2017 [3] and spiked with 0.1 to 0.9 ng/mL of the explosives. The second set of mussels samples was prepared using the freeze-drying and solid-phase extraction method and was spiked from 0.01 to 0.09 ng/mL, as well as from 0.1 to 0.9 ng/mL. The detection limits for all samples were determined using the splitless quartz wool liner method. Additionally, for the freeze-dried mussels, the programmable temperature vaporization—large volume method (PTV-LVI) method was tested.

## 3. Results

### 3.1. GC-MS/MS Method Development and Optimization

**Figure 1:**
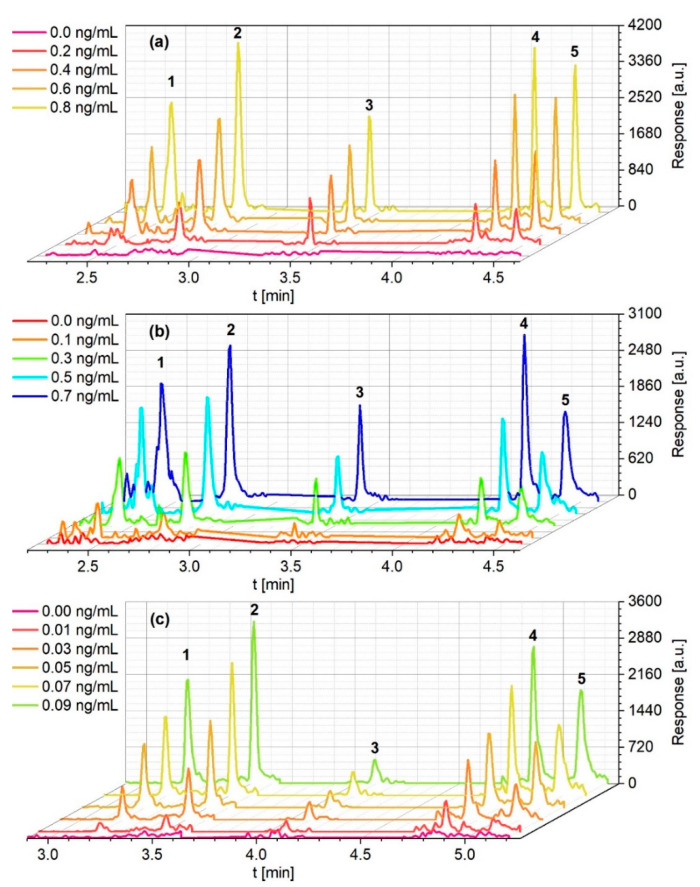
Chromatograms of the calibration standards used to determine the limits of detection (LODs) of the (**a**) splitless quartz wool (**b**) splitless CarboFrit and (**c**) programmable temperature vaporization—large volume injection methods. Peaks are 1: 1,3-dinitrobenzene (1,3-DNB); 2: 2,4-dinitrotoluene (2,4-DNT); 3: TNT; 4: 4-amino-2,6-dinitrotoluene (4-ADNT); 5: 2-amino-2,4-dinitrotoluene (2-ADNT).

The mass spectrometer was operated in secondary reaction monitoring mode, as this allows reliable detection of the substances even at very low concentrations. The quantitative and qualitative transitions observed, and the corresponding collision energies are also presented in Table 2. The instrumental Limits of Detection determined for both methods can be found in Table 3. Figure 1 shows the chromatograms of different concentrations used to determine them.

#### 3.1.1. Splitless Injection Methods

The splitless technique allows injection of up to 1 µL without the use of a retention gap. In splitless injection, the sample is injected into the liner at a temperature well above the boiling point of the solvent. Irrespective of their boiling points, all analytes are vaporized at the same temperature immediately after injection. 

**Figure 2:**
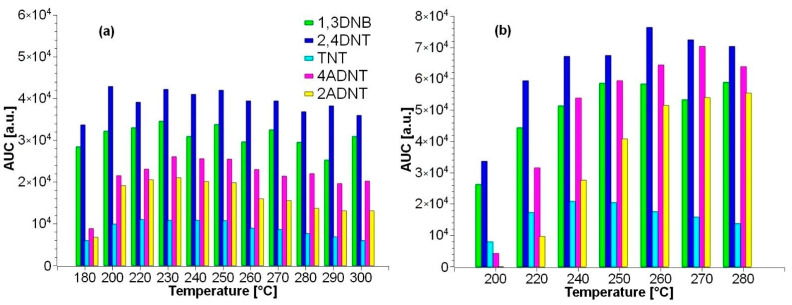
Temperature optimization for splitless injection using (**a**) packed quartz wool and (**b**) CarboFrit injection port liners. The optimum temperature was determined to be 230 °C for the quartz wool and 270 °C for the CarboFrit liner.

To determine the optimal injector temperature, 1 µL of a 10 ng/mL mixture of 1,3-DNB, 2,4-DNT, TNT, 4-ADNT, and 2-ADNT was injected into a splitless packed quartz wool liner at various temperatures between 180 °C and 300 °C, and the peak areas of the compounds were recorded. Thus, 230 °C was determined as the optimum injection temperature, as shown Figure 2a. According to Emmrich et al. 2001 [21], the use of packed CarboFrit liner instead of quartz wool is highly recommended. The same packing material was used by Marder et al. (2018) [22]. Initial experiments with an injector temperature of 290 °C almost resulted in a complete loss of the TNT peak from the second injection onwards, so temperatures between 180 and 280 °C were tested. The highest intensities were obtained at a temperature of 270 °C (Figure 2b). Although initial tests looked promising, CarboFit liners did not provide a clear advantage over the more favorable quartz wool liners within the detection limits (Table 3).

Since many of the explosives are temperature sensitive (e.g., TNT starts to decompose around 150 °C [23,24]), the dwell time on the column was minimized by a rapid oven program, and the 15 m column was used instead of 30 m. Thermo Fisher Scientific TG-5MS amine columns, which are specially deactivated to reduce tailing of amines such as 2- and 4-ADNT, were found to provide better results compared to standard 5% diphenyl/95% dimethyl-polysiloxan phases. Columns were trimmed by 5 cm each time the liner was exchanged.

The initial oven temperature was set to 100 °C to prevent condensation of the solvent on the column. Higher initial temperatures resulted in a peak broadening of 1,3-DNB. After a 0.20 min condensation phase, the oven temperature was increased by 30 °C/min. This was the highest rate at which 4- and 2-ADNT could still be clearly separated. The retention times are between 2.43 min (1,3-DNB) and 4.42 min (2-ADNT), as presented in Table 2. After 2-ADNT has left the column, it was heated to 280 °C at 80 °C/min and held for one minute to remove low volatile compounds. Thus, a total runtime of only 6.25 min could be achieved.

#### 3.1.2. PTV Large Volume Injection Method

Different types of liners were tested; packed quartz wool, CarboFrit, baffled glass, and sintered glass. Although the latter allows the highest injection volumes; in preliminary tests, up to 50 µL were tested with standards in acetonitrile, these liners are extremely susceptible to contamination by matrix components due to the dense glass mesh. Initial tests with spiked water and mussel samples showed a rapid decrease in peak intensities after only a few injections, even when the injection volume was reduced to 5 µL. This was accompanied by a clearly visible discoloration of the liner. They were, therefore, not used further for method development. According to the manufacturer, baffled liners are suitable for volumes up to 10 µL; however, no satisfactory results could be obtained here. After a few measurements, strong turbidity of the column end could be observed, which indicates insufficient retainment of the sample solution in the liner before evaporation. The best results were obtained with quartz wool which, however, limits the injection volume to <10 µL.

Five µL was chosen as the best compromise between detection limit and wear. A solvent split of 0.18 min at 70 °C and 50 mL/min split flow was set at the beginning of the injector heating program, followed by a 5 °C/s temperature increase to a final temperature of 240 °C. The remaining low volatile compounds were removed from the liner by 200 mL/min split-flow 1.5 min after the final temperature was reached. 

The oven program of the splitless method was adapted. Due to the solvent split, the initial time at 100 °C was increased from 0.2 to one minute. The heating rate was set for 35 °C/min to 220 °C (0.7 min), and by 70 °C/min to 280 °C. Total run time was just under 7 min. Due to the solvent split at the beginning of the heating program, the retention times in the PTV method were somewhat longer than the splitless method. They are between 3.20 min (1,3-DNB) and 5.07 min (2-ADNT) and can be found in Table 2. Instrumental Limits of Detection of the PTV-LVI method can be found in Table 3; Figure 1c shows the chromatograms from 0.00 to 0.09 ng/mL which were used to determine them.

### 3.2. Improvement of Sample Preparation

#### 3.2.1. Water Samples

In previous studies, conducted directly at the surface of exposed explosives, TNT concentrations of more than 3 mg/L were measured, but these concentrations decreased to 3.3 µg/L at a distance of only 50 cm from the exposure site [25]. In open water, concentrations ranging from <0.5–>7.1 ng/L were detected in a large number of water samples collected from 114 stations during a research cruise along the German Baltic coast, with the highest concentrations found in nearshore water and near the bottom [26].

The method of water sampling was adopted from Gledhill et al. 2019 [19] and adapted slightly. In the current study, bags were connected to the upper end of the column, via a homemade adapter instead of the Luer lock connection at the bottom, and the eluate was concentrated to 600 µL in vacuum.

**Figure 3:**
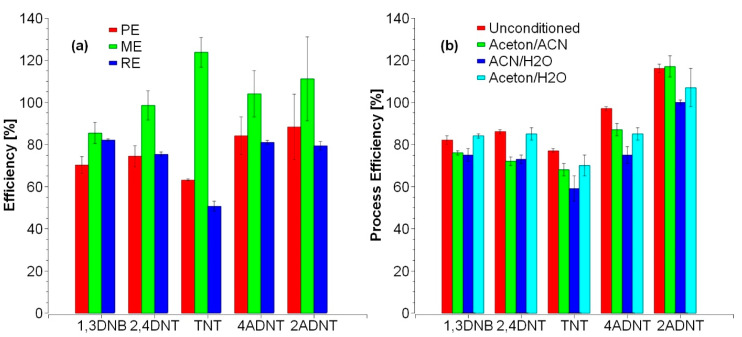
(**a**) Process efficiency (PE; *n* = 6), matrix effect (ME; *n* = 6), and recovery efficiency (RE, *n* = 2) of 10 ng of the mentioned explosives by SPE-Extraction with unconditioned Chromabond Easy columns. (**b**) Process efficiency of different column conditions. No major advantages were found compared to the unconditioned ones.

The effect of preconditioning on the performance of columns, as mentioned in the Macherey Nagel application database, was tsted; since manufacturers advertised these columns as ready-to-use. Preconditioning with acetone followed by water, ACN followed by water, and acetone followed by ACN were tested against the unconditioned columns in terms of process efficiency by extracting 10 ng of each explosive from 1 L ultrapure water. Process efficiency, matrix effect, and recovery efficiency were determined for the unconditioned columns (Figure 3a). No major advantage was found for the conditioning step regarding the process efficiency (Figure 3b).

#### 3.2.2. Sediment Samples

**Figure 4:**
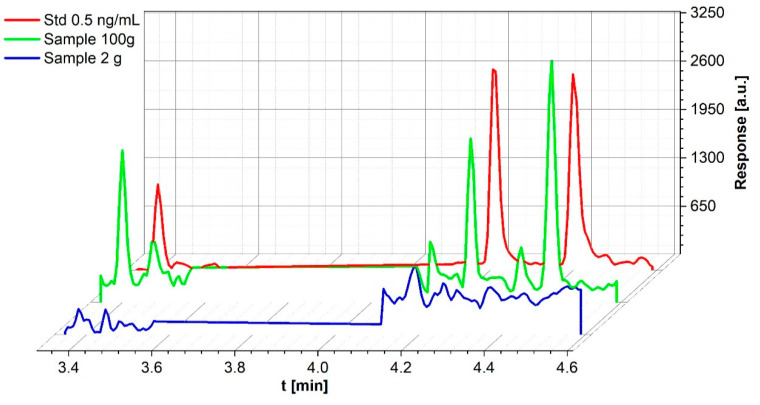
Comparison of the same sediment sample once processed with 2 g freeze-dried material and once with 100 g wet sediment with a 0.5 ng/mL standard. While no explosives were found in the lyophilized sample, 0.6 ng TNT (1), 0.3 ng 4-ADNT (2), and 0.5 ng 2-ADNT (3) were detected in the wet one.

For sediment samples, the method published in the UDEMM Best Practice Guide [27] was initially used. Twenty grams of sediment were measured and freeze-dried. The average weight lost during drying was 24.9 ± 3.8% (*n* = 18). From the homogenated samples, 2 g were weighed for preparation. Previous tests have shown that explosive concentrations in sediments are often very low, and occur only when sediment and munitions are in direct contact. They can be found in the samples. Even in an area with TNT concentrations of more than 50 pg/g water, sediment concentrations were below 1 pg/g. Therefore, in order to detect even lower amounts of explosives in sediment samples, a method was developed using solid-phase extraction for 100 g non-freeze-dried sediment. With this method, explosive concentrations of less than 10 pg/g sediment could be detected in samples collected close to sunken warships. TNT (0.6 ng/mL, corresponding to 6 pg/g weight), 4-ADNT (0.3 ng/mL, 3 pg/g weight), and 2-ADNT (0.5 ng/mL, 5 pg/g weight) were detected in a sample in which no explosives were measured in 2 g of freeze-dried material (Figure 4).

#### 3.2.3. Mussel Samples

While the method of mussel preparation published by Strehse et al. 2017 [3] was capable of proving the accumulation of TNT and its metabolites and allows quick and simple preparation of large quantities of samples, its limits of detection were relatively high (1.2–3.5 ng/g wet weight). This was because the mussels were homogenized as a whole and thus contained large quantities of water. Moreover, samples were then aliquoted into 1 g portions in 10 mL of ACN, thus equaling only 0.1 g of whole mussel tissue per mL. In open water experiments with mussels transplanted in the dumping area Kolberger Heide, concentrations of up to 31.0 ng/g w.w. TNT, 131.3 ng/g w.w. 4-ADNT, and 103.8 ng/g w.w. 2-ADNT were recorded in mussels after 93 days of exposure, which were placed directly on free-lying hexanite (German: “Schiesswolle”) [3], while in mussels exposed in similar time frames in the vicinity of corroding mines only 4-ADNT could be detected at concentrations of 2.4–7.8 ng/g w.w. [4]. Furthermore, a near-linear decrease in 4-ADNT concentration was observed; between 10 cm and 1 m vertical distance, it had decreased by almost 90% [5]. Therefore, for mussels placed in open water from less polluted areas, 4-ADNT concentrations well below 1 ng/g w.w. can be expected.

To achieve lower limits of detection, the water was gently removed from the mussel tissue by freeze-drying. The average dry weight (d.w.) was determined as 10.0 ± 2.1% of the wet weight (w.w.) (*n* = 14). Approximately 250 mg of the freeze-dried tissue was extracted in 2 mL ACN by means of an ultrasonic bath. The samples were analyzed using the 5 µL large volume injection programmable temperature vaporization method. Coeluted polar mussel compounds such as fatty acids, sterols, etc., however, resulted in a rapid decrease in sensitivity such that a 1 ng/mL standard mix was not detected after measuring less than 10 samples.

Polytetrafluoroethylene (PTFE) filters, as recommended by Bae et al. [28], were tested to reduce the buildup of coarse mussel debris on the liners, with process efficiencies slightly above 100% for 0.22 and 0.45 µm pore sizes (mussel samples were spiked with 50 or 100 ng/mL of the standard mixture, *n* = 3).

**Figure 5:**
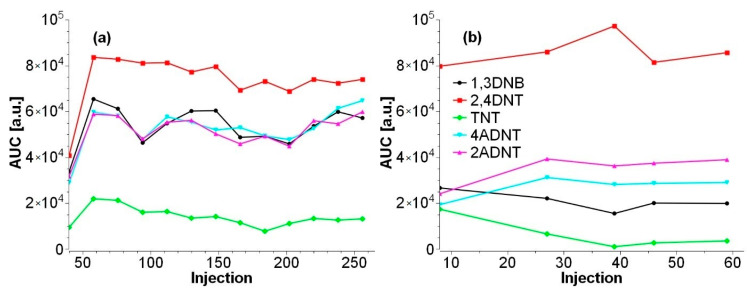
Course of the peak area of an external standard of (**a**) 10 ng/mL injected after every 16 measurements for a batch of 220 mussel samples prepared according to Strehse et al. 2017 and measured by splitless injection, and (**b**) 1 ng/mL 50 freeze-dried/SPE extracted samples measured by PTV-LVI. The intensity of TNT decreased by 50% (splitless) and 90% (PTV-LVI) during measurements, while the intensities of the other explosives increased slightly after the first measurements and then remained at the same level. For the splitless method, ^13^C^15^N-TNT was used as an internal standard to correct the area of TNT.

Furthermore, a solid phase extraction method was developed to separate the explosives from the matrix. An amount of 250 to 500 mg mussel tissue was extracted in 2 mL ACN as described above. After centrifugation, the supernatant was made up to 25 mL in ultrapure water and concentrated by solid-phase extraction. The eluate was concentrated to 600 µL. Freeze-drying prior to processing reduced the detection limits in the mussel samples by a factor of 100, and even by a factor of 1000 in combination with solid-phase extraction (Table 4). The reliability of both methods was tested and confirmed using calibration standards in a series of measurements of actual samples (Figure 5).

#### 3.2.4. Limits of Detection/-Quantification and Linear Range

Limits of detection (LOD) and—quantification (LOQ) of the methods were determined for solvent standards in ACN, water, sediment, and mussels (fresh and lyophilized) by splitless injection on quartz wool liners (SL QW), and for solvent standards as well as lyophilized mussels by the programmable temperature vaporization large volume injection method (PTV LVI). The calibration standard method according to EUR 28,099 EN was used [29]. Matrix blank samples were spiked with a mixture of the analytes in five equidistant steps, from zero to ten times the estimated limit of detection. For each concentration, two independent samples were prepared. The LOD was calculated in an excel sheet, and LOQ was defined as 3.3 times the LOD.

For the SL QW-method, LODs between 95 and 333 fg/µL (equals the quantity on the column) were achieved. Similar or minimally lower detection limits were observed using CarboFrit liners. For the PTV large volume injection method, limits were 3 times (TNT, 47 fg/µL, 235 fg on column) to 12 times (4-ADNT, 8 fg/µL, 40 fg on column) lower. LODs and LOQs for standards prepared in ACN are displayed in Table 3. Linearity was determined for all components between the LOQ and 500 ng/mL (R^2^ ≥ 0.995).

Due to the small differences between CarboFrit and quartz wool liner, matrix-specific LODs were determined only for the latter. The values obtained were divided by the amount of sample used and thus converted to ng/L or ng/g (w.w. or d.w). The calculation for sediments refers to the extraction of 2 g of lyophilized material. With the extraction of 100 g wet sediment using the solid phase method, 30–50 times lower detection limits could be achieved. Comparing fresh mussels with freeze-dried ones, taking into account the 90% weight loss due to freeze-drying, detection limits achieved for 2- and 4-ADNT were 100-fold lower using SL QW and almost a thousand-fold lower using PTV-LVI. Matrix-specific LODs and LOQs can be found in Table 4.

## 4. Discussion

Compared to split-/splitless injectors, programmable temperature vaporization injectors have some advantages. With programmable temperature vaporization injectors, the sample is applied to the liner at temperatures below the boiling point of the solvent, which is then heated to a target temperature at a rate of a few degrees per second. Thus, each substance is vaporized at its precise boiling point, and this protects temperature-labile analytes. During this process, the solvent can be selectively evaporated and larger amounts of the analyte can be injected into the liner (up to several 100 µL). However, its disadvantages are the significantly lower liner volumes required (which lead to faster contamination of the packing material by low-volatility matrix components), and the significantly high cost of the liners.

The methods established in this study allow sample- and concentration-dependent preparation, and detection of the five main legacy explosive chemicals from marine-dumped munitions. In accordance with the expected concentration and desired limits of detection, work-up is fast, and with the addition of further cleaning steps such as solid-phase extraction, even the lowest concentrations can be determined. Detection limits from a few tens to a few hundreds of femtograms per microliter could be achieved in all matrices tested.

Compared to Strehse et al., 2017 [3], a combination of freeze-drying and solid-phase extraction allowed a hundred times lower detection limits for 4- and 2-ADNT in mussels when analyzed by splitless injection. These detection limits recorded could also be a thousand times lower when using the PTV injector.

Moreover, this method is excellent for rapid and reliable analysis of large amounts of samples and, with the improvements developed here, allows detection limits between 1.2 and 3.5 ng/g wet weight (equivalent to 12–35 ng/g dry weight). Importantly for both methods, their suitability was proven on large measurement series, as shown in Figure 5.

In comparison with other GC- and LC-MS methods reported in recent years, the detection limits achieved in this study are among the lowest published so far (Table 5). They are more than one order of magnitude lower than those described by Gordon et al. 2018 [20] and Dawidziuk et al. 2018 [30]. Moreover, with the exception of TNT, these detection limits are even more sensitive than those found by Gledhill et al. 2019 [19] using UHPLC-Orbitrap-MS. Only Kirchner et al. 2007 [31] yielded a lower detection limit for TNT and a comparable one for 2,4-DNT. However, their measurements were performed by on-column injection onto a very short column of 5 m, which is likely to lead to very rapid contamination due to the complex matrices in the marine samples. Furthermore, their detection limits were determined from the signal-to-noise ratios, which makes the comparability between the methods difficult.

## 5. Conclusions

With the methods presented in this study, reliable, fast, and sensitive determination of low explosive concentrations in marine samples, such as water, sediment, and marine biota are possible. The splitless injection method allows rapid measurement in only 6.25 min with limits of detection between 77 to 333 fg/µL. Combined with the applied extraction and sample preparation methods, it can be adapted for multiple types of biotic and abiotic samples. For biota samples (e.g., *Mytilus* spp.), in which the parent TNT concentration is lower compared to its metabolites, the large volume injection method allows detection limits that are even 10 times lower (of note: 8–47 fg/µL, corresponds 40 to 235 µg on column). Altogether, the methods described in the present investigation are among those with the lowest detection limits published to date and have successfully been proven for their reliability on actual samples, i.e., samples obtained from different scientific research projects [3,4,5,6]. Furthermore, for the splitless method, using over 200 samples, the areas of an external 10 ng/mL standard remained constant for 1,3-DNB, 2,4-DNT, 2- and 4-ADNT, and only decreased by 50% for TNT. This allows automatic measurements over several days. The PTV method has been proven over a batch with 50 samples and has provided consistent results for 2- and 4-ADNT over the entire course of measurement.

## Figures and Tables

**Table 1:** GC-MS/MS programs for splitless and large volume injections.

Parameter	Splitless		Large Volume Injection
Injector	Split-/splitless		Programmable temp. vaporization
Inlet liner	Quartz wool	CarboFrit	Quartz wool
Injection volume	1 µL		5 µL
Injection temperature	230 °C	270 °C	70 °C, (0.18 min, 50 mL × min^−1^) 5 °C/s to 240 °C (1.5 min, no split) 240 °C (5 min, 200 mL/min^−1^)
Column flow	1.5 mL × min^−1^		1.2 mL × min^−1^
Oven temp.	100 °C (0.20 min), 30 °C/min to 220 °C (0.30 min), 80 °C to 280 °C (1 min)		100 °C (1 min), 35 °C/min to 220 °C (0.7 min), 70 °C to 280 °C (1 min)
Total run time	6.25 min		6.99 min
Transfer line temp.	250 °C		
Ion source temp.	300 °C		
Ionization method	EI		

**Table 2:** Retention times for the splitless (Rt SL) and programmable temperature vaporization (PTV)-large volume injection (Rt LVI) methods; quantitative (Q) and qualitative (q) secondary reaction monitoring transitions (m/z) of explosives and internal standard ^13^C^15^N-2,4,6-trinitrotoluene (TNT).

Compound	Rt SL [min]	Rt LVI [min]	Molecular Mass [g × mol^−1^]	Transition [m/z]		CE [eV]
1,3-Dinitrobenzene	2.43	3.20	168.11	Q	122.0 > 75.0	12
				q	168.0 > 75.0	20
				q	168.0 > 122.0	8
2,4-Dinitrotoluene	2.77	3.52	182.13	Q	165.0 > 63.1	22
				q	165.0 > 90.1	16
				q	165.0 > 118.1	8
Trinitrotoluene	3.41	4.09	227.13	Q	210.0 > 164.1	6
				q	164.0 > 90.1	10
				q	108.0 > 76.1	12
^13^C^15^N-Trinitrotoluene	3.41	4.09	237.06	Q	220.1 > 173.1	6
				q	220.1 > 203.1	8
				q	189.1 > 82.1	10
4-Amino-2,6-dinitrotoluene	4.22	4.85	197.15	Q	197.0 > 180.1	6
				q	180.0 > 163.1	8
				q	163.0 > 78.0	14
2-Amino-4,6-dinitrotoluene	4.42	5.07	197.15	Q	197.0 > 180.1	6
				q	180.0 > 133.0	6
				q	180.0 > 67.0	12

**Table 3:** Detection and quantification limits of the applied methods. On column, volumes are 1 µL for the splitless and 5 µL for the PTV large-volume method.

Compound	SL QW			Splitless, CarboFrit Liner			PTV-LVI		
	LOD LOQ		R^2^	LOD LOQ		R^2^	LOD LOQ		R^2^
	fg/µL			fg/µL			fg/µL		
1,3-DNB	333	1099	0.9444	330	1089	0.9664	32	105	0.9644
2,4-DNT	77	254	0.9968	86	284	0.9951	10	33	0.9934
TNT	152	502	0.9879	150	495	0.9852	47	155	0.9878
4-ADNT	95	314	0.9951	88	289	0.9896	8	26	0.9959
2-ADNT	103	341	0.9943	60	210	0.9944	11	37	0.9919

**Table 4:** Matrix-specific limits of detection of the methods developed for water, sediment, and mussel samples.

Compound	Water			Sediment			Mussels by Strehse et al. 2017 [3]			Freeze Dried Mussels by Solid Phase Extraction					
	SL QW												PTV LVI		
	LOD LOQ ng/L		R^2^	LOD LOQ ng/g d.w.		R^2^	LOD LOQ ng/g w.w.		R^2^	LOD LOQ ng/g d.w.		R^2^	LOD LOQ ng/g d.w.		R^2^
1,3DNB	0.20	0.66	0.978	0.10	0.33	0.944	2.2	7.2	0.975	0.27	0.88	0.977	0.03	0.10	0.985
2,4DNT	0.05	0.15	0.998	0.02	0.08	0.996	2.2	7.3	0.975	0.23	0.75	0.983	0.04	0.12	0.990
TNT	0.09	0.30	0.981	0.05	0.15	0.988	3.5	11.5	0.940	0.47	1.60	0.964	0.20	0.68	0.986
4ADNT	0.06	0.19	0.994	0.03	0.09	0.995	1.5	5.1	0.987	0.27	0.90	0.988	0.05	0.17	0.973
2ADNT	0.06	0.20	0.992	0.03	0.10	0.994	1.2	4.0	0.992	0.10	0.32	0.999	0.04	0.14	0.980

**Table 5:** Comparison of the instrumental detection limits (pg/µL) achieved in this study with those published by other authors.

	Present Study		Gledhill et al. 2019 [19]	Gordon et al. 2018 [20]	Dawidziuk et al. 2018 [30]	Kirchner et al. 2007 [31]
Method	SL QW	PTV LVI	LC-Orbitrap	GC-MS/MS	GC-MS/MS	GC-MS
	pg/µL					
1,3-DNB	0.333	0.032	0.15	3.6	4.8	
2,4-DNT	0.077	0.010	1.05	2.7	4.3	0.063
TNT	0.152	0.047	0.036	560.4	19.9	0.029
4-ADNT	0.095	0.008	0.050	47		
2-ADNT	0.103	0.011	0.030	13.1		

## Data Availability

Data are contained within the article.
